# Effects of 7‐minute practices of breathing and meditation on stress reduction

**DOI:** 10.1002/pchj.702

**Published:** 2023-11-09

**Authors:** Chunxia Sun, Jiajin Tong, Xin Qi, Zhonghui He, Junwei Qian

**Affiliations:** ^1^ Department of Management and Social Psychology, School of Psychological and Cognitive Sciences, Beijing Key Laboratory of Behavior and Mental Health Peking University Beijing China; ^2^ Department of Physical Education and Research Peking University Beijing China

**Keywords:** breathing, low‐dose intervention, meditation, micro‐breaks, perceived stress reduction

## Abstract

We compared the effects of 7‐min practices of breathing and meditation on perceived stress reduction and related affective outcomes (active emotion, serenity, anxiety, and fatigue) during micro‐breaks. Undergraduates from two classes (*N* = 59) completed the 7‐point online surveys. Results supported the effects of both practices.

An increasing number of college students experience high levels of stress, which adversely affects their emotional well‐being, academic performance, and physical health (Yusufov et al., 2019). Various stress reduction interventions have been used among students, and researchers have explored the potential differences between multiple interventions (e.g., breathing and meditation; Qi et al., 2020) to guide students in choosing a feasible and effective practice to cope with stress. However, few studies have explored and compared the immediate effects of low‐dose interventions, such as short‐time interventions during daily micro‐breaks. To maximize practicality, we examined and compared the effects of one‐time breathing and meditation practices on perceived stress reduction during a 7‐min break for undergraduates.

Findings from studies investigating the durable effects of breathing and meditation on students have demonstrated significant improvements in psychological health and in academic achievement (Paul et al., 2007). Matsuura and Yamazaki (2021) found that a regular practice of controlled breathing resulted in stress reduction, with the primary benefits being restored energy and improved mood states. Meditation can also be used to manage stressful situations, as it is a technique for developing awareness and attention and achieving calmness (Bishop et al., 2004). Additionally, emotions and emotion regulation are closely related to coping with stress (Wang & Saudino, 2011), so the study of affective changes may be a good way to understand the process of stress reduction. Taken together, we expected that breathing and meditation would have a significant effect on perceived stress reduction and related affective outcomes.

We collected data from two classes of undergraduate students in a Chinese university. This quasi‐experimental study followed a within‐group design, with all subjects (*N* = 59) taking three types of micro‐breaks successively in the order of control, breathing, and meditation. Each type of intervention was conducted only once. The interventions were given aurally and instructed participants to engage in breathing (“breathe slowly and deeply”) and meditation (“imagine some pictures…gaze at the picture in detail and keep focused…”) practices, as conducted in previous research (e.g., Qi et al., 2020). Interventions lasted less than 7 min for each micro‐break, between two sessions of 50‐min regular classes for applied psychology in three consecutive weeks. The course content was not related to the interventions. Participants scanned QR codes to fill out the pre‐ and post‐intervention surveys. The surveys were the same for all three micro‐breaks. Data were collected via online surveys, with voluntary participation. As in previous research (e.g., Qi et al., 2020), perceived stress for the present moment was measured by seven items adopted from the Stress subscale in the Depression Anxiety Stress Scale (Lovibond & Lovibond, 1995), ranging from 1 (*Never*) to 7 (*Always*). Stress‐related emotions were adapted from the Positive and Negative Affect Schedule‐Expanded Form (Watson & Clark, 1994), ranging from 1 (*Not at all*) to 7 (*Extremely*). Active emotion (“active”, “excited” and “inspired”), serenity (“placid”, “calm” and “relaxed”), anxiety (“anxious” and “nervous”), and fatigue (“fatigued”, “drowsy” and “tired”) were chosen because they have been frequently explored in previous stress research and cover both positive and negative emotions ranging from weak to strong.

The sample size was determined using G*Power 3.1 (*α* = 0.05, 1 – *β* = 0.9, effect size = 0.25, #group = 1, #measurements = 6), which indicated that a minimum of 24 participants were required. Because the within‐group design may underestimate the sample size, we recruited 59 undergraduate students. Participants ranged from 18 to 23 years old, with an average age of 20.43 (*SD* = 1.05) and included 22 males and 37 females. To investigate the effects of breathing and meditation, the study employed a 3 (intervention: control, breathing, meditation) × 2 (time: pretest, posttest) design, and a repeated‐measures analysis of variance (ANOVA) was conducted using the Bonferroni method for multiple comparisons. For perceived stress, the interaction of interventions and time (pre‐post intervention) was significant (*F*[2,57]) = 8.17, *p* < .001, η_p_^2^ = .22). Specifically, the pretest scores were significantly higher than the posttest scores for breathing (Δ*M* = −0.436, *SE* = 0.09, *p* < .001) and meditation (Δ*M* = −0.547, *SE* = 0.11, *p* < .001), but not for the control condition (Δ*M* = 0.06, *SE* = 0.10, *p* = .54).

The following analysis revealed the effects of interventions on stress‐related affective outcomes. The interactions of interventions and time were significant for serenity (*F*[2,57] = 7.56, *p* = .001, η_p_^2^ = .22) and anxiety (*F*[2,57] = 5.61, *p* = .006, η_p_^2^ = .16). But the interactions were marginal for fatigue (*F*[2,57] = 3.07, *p* = .054, η_p_^2^ = .10) and not significant for active (*F*[2,57] = 1.30, *p* = .28, η_p_^2^ = .04). In more detail, in terms of serenity and anxiety, the changes from the pretest to the posttest scores were significant for breathing (Δ*M*_serenity_ = 0.31, *SE* = 0.08, *p* < .001; Δ*M*_anxiety_ = −0.75, *SE* = 0.15, *p* < .001) and meditation (Δ*M*_serenity_ = 0.52, *SE* = 0.10, *p* < .001; Δ*M*_anxiety_ = −0.44, *SE* = 0.18, *p* = .02), but not for the control condition (Δ*M*_serenity_ = 0.05, *SE* = 0.08, *p* = .59; Δ*M*_anxiety_ = −0.16, *SE* = 0.12, *p* = .19). For active and fatigue, in contrast to the control condition (Δ*M*_fatigue_ <0.01, SE = 0.10, *p* = 1; Δ*M*_active_ = 0.12, *SE* = 0.09, *p* = .19), the changes from the pretest to the posttest scores were significant for breathing (Δ*M*_active_ = 0.30, *SE* = 0.10, *p* = .003; Δ*M*_fatigue_ = −0.34, *SE* = 0.13, *p* = .008) and for meditation (Δ*M*_active_ = 0.24, *SE* = 0.11, *p* = .037), but the main effect of time was not significant for meditation in predicting fatigue (Δ*M*_fatigue_ = −0.27, *SE* = 0.16, *p* = .10).

The results showed that breathing and meditation practices as short as 7 min can have an immediate impact on reducing perceived stress. However, they function in different ways. The distinguishing effect of breathing is to elevate positive emotions (serenity and active emotion) and decrease negative emotions (anxiety and fatigue), while meditation mainly develops a state of calmness and concentration by increasing positive emotions (serenity and active emotion) and decreasing negative emotions (anxiety). These results are consistent with previous literature that shows that breathing reduces stress mainly by suppressing negative energy‐sapping emotions so that practitioners become calm and energetic (Matsuura & Yamazaki, 2021). In terms of meditation, the previous literature indicated that meditation requires consciously focusing on current internal and external experiences in a non‐judgmental and accepting way (Bishop et al., 2004). Thus, meditation may not help in the reduction of negative emotions such as fatigue, but it can reduce stress by promoting the acceptance of negative situations with serenity. Because they can be effective at low‐dose levels, breathing and meditation are effective ways for students to cope with daily stress and regain energy instantly. They could be used to deal with different types of stressors owing to their potential differences, a topic that deserves future research. Further, longer‐term follow‐up measurements may provide valuable insights into the sustainability of the observed effects.

## CONFLICT OF INTEREST STATEMENT

The authors declare there are no conflicts of interest.

## ETHICS STATEMENT

All participants provided written informed consent. The study was approved by the Institutional Review Board of Peking University.

## Data Availability

Data from this work are available through the corresponding author upon reasonable request.
